# RATIONALE FOR ASPREE DISABILITY-FREE SURVIVAL PRIMARY OUTCOME AND OVERVIEW OF PRIMARY OUTCOME RESULTS

**DOI:** 10.1093/geroni/igz038.2357

**Published:** 2019-11-08

**Authors:** John McNeil

**Affiliations:** Department of Epidemiology & Preventive Medicine, Monash University, Melbourne VIC, Australia, Melbourne VIC, Australia, Australia

## Abstract

Disability-free survival (DFS), defined as survival free of disability and dementia was the primary outcome measure of the ASPREE clinical trial. As previously reported, there was no benefit of low dose aspirin on the primary end point of dementia, physical disability or death, but bleeding risks were increased. In total, 1,835 participants reached the primary endpoint, confirmed amongst approximately 3,000 who had triggered for one of the end-points. Dementia was the most labor intensive component of DFS. Several previous primary prevention aspirin studies had identified a reduction of vascular events counterbalanced by an increase in serious bleeding, leaving the question of net outcome to an intuitive decision. DFS was chosen because it balances the positive and negative effects of a preventive drug such as aspirin. It also encapsulates the primary purpose of a preventive drug in older people i.e., to prolong a healthy lifespan rather than prevent a defined disease.

